# Interferon-alpha-Induced Changes in NODDI Predispose to the Development of Fatigue

**DOI:** 10.1016/j.neuroscience.2017.12.040

**Published:** 2019-04-01

**Authors:** N.G. Dowell, S. Bouyagoub, J. Tibble, V. Voon, M. Cercignani, N.A. Harrison

**Affiliations:** a Department of Neuroscience, Brighton and Sussex Medical School, University of Sussex, Brighton BN1 9RR, UK; b Department of Gastroenterology, Brighton & Sussex University Hospitals, Brighton, UK; c Department of Psychiatry, University of Cambridge, Cambridge CB2 0QQ, UK; d Neuroimaging Laboratory, Santa Lucia Foundation, Rome, Italy; e Sackler Centre for Consciousness Science, University of Sussex, Falmer BN1 9RR, UK; f Sussex Partnership NHS Foundation Trust, Brighton, UK

**Keywords:** FDG, fluorodeoxyglucose, FWE, Family-Wise Error, HAMD, Hamilton Depression Rating Scale, IFN-α, interferon-alpha, MRI, magnetic resonance imaging, MT, magnetization transfer, NDI, neurite density index, NODDI, neurite orientation dispersion and density imaging, ODI, orientation-dispersion index, ROI, regions of interest, TNF, tumor necrosis factor, cytokine, depression, diffusion MRI, fatigue, inflammation

## Abstract

Interferon-alpha (IFN-α) is an important mediator of antiviral immune responses. It is also used clinically in the treatment of hepatitis-C infection. Though effective, IFN-α-based therapies can often impair mood, motivation and cognition, which when severe can appear indistinguishable from major depression. In susceptible patients, fatigue and motivational impairment emerge early and have been linked to changes in basal ganglia (striatal) metabolism, neurochemistry and microstructural integrity. Here we use neurite orientation dispersion and density imaging (NODDI) modeling of multi-shell diffusion MRI to investigate whether changes in orientation-dispersion index (ODI) or neurite density index (NDI) can predict the later emergence of IFN-α-induced fatigue. Eighteen patients initiating IFN-α-based treatment for hepatitis-C underwent diffusion MRI and blood sampling at baseline and 4 h after their first IFN-α injection. They were then followed up with regular psychological assessments for 12 weeks of treatment. IFN-α injection stimulated an acute inflammatory cytokine response and evoked acute fatigue that peaked between 4 and 12 weeks of treatment. Within the brain, IFN-α induced an acute increase in NDI in patients that experienced a simultaneous increase in IFN-α-induced fatigue but not in patients that did not. Acute changes in striatal microstructure additionally predicted the continued development of fatigue but not mood symptoms 4 and 8 weeks later into treatment. Our findings highlight the value of NODDI as a potential *in vivo* biomarker of the central effects of peripheral inflammation. We highlight the exquisite sensitivity of the striatum to IFN-α and further implicate striatal perturbation in IFN-α-induced fatigue.

## Highlights

• NODDI, was used to probe subtle changes to tissue microstructure associated with IFN-α.
• We found a strong correlation between changes in neurite density index with acute and long-term fatigue following IFN-α.
• This observation confirms that the striatum is a key brain area targeted by IFN with implications for impaired motivation.

## Introduction

Interferon-alpha (IFN-α) is a type I interferon that is released by specialized immune cells (Liu, 2005) and virally infected cells and promotes broad antiviral immune responses. These anti-viral properties of IFN-α are harnessed clinically in the treatment of Hepatitis-C infection. However, despite its good clinical efficacy, direct and/or indirect actions of IFN-α on the brain frequently result in highly disabling behavioral changes including fatigue, motivation, mood, and cognitive impairments (Capuron et al., 2002). When severe, these changes can appear indistinguishable from major depression and provide powerful support for inflammatory theories of depression (Musselman et al., 2001, Bonaccorso et al., 2001, Dantzer et al., 2008). Symptoms such as fatigue, psychomotor slowing and motivation change typically emerge rapidly, often within hours of the first IFN-α injection suggesting that IFN-α can rapidly engage immune–brain communicatory pathways (Capuron et al., 2002, Dowell et al., 2016).

Unlike inflammation induced using bacterial antigens (Rosenkranz et al., 2005, Harrison et al., 2009, Harrison et al., 2015, Lekander et al., 2016), the imaging literature provides limited evidence that the central effects of IFN-α result from engagement of visceral afferents and interoceptive pathways to insula (Capuron et al., 2007, Capuron et al., 2012, Haroon et al., 2014, Dowell et al., 2016). Instead, IFN-α appears to exert its effects predominantly through direct actions on the brain. The blood–brain interface (BBI) is permeable to IFN-α with greater permeability observed in sub-cortical than cortical regions (Pan et al., 1997, Banks, 2016). In rodents, peripheral IFN-α also leads to rapid up-regulation of interferon-sensitive genes with subcortical areas (Wang and Campbell, 2005, Wang et al., 2008). This sensitivity of sub-cortical and particularly striatal structures to INF-α is also observed in human clinical and imaging studies (Lebon et al., 1988). For example, chronic IFN-α administration is associated with bilateral (though left predominant) increases in striatal ^18^fluoro-deoxy-glucose (^18^FDG) uptake (Capuron et al., 2012), left-, but not right-sided, increases in striatal glutamate/creatine ratio (Haroon et al., 2014); and increased striatal ^18^fluorodopa uptake (Capuron et al., 2012). Recently, we have also demonstrated that IFN-α results in rapid (within 4 h) changes in bilateral (though left predominant) striatal magnetization transfer (MT), specifically an increase in MT from free (water) to molecular-bound protons (*k*_f_) and a complementary reduction in free water spin–spin relaxation time (T_2f_) (Dowell et al., 2016). Across each of these studies striatal changes correlate with between-subject sensitivity to IFN-α-induced motivational change and/or subjective fatigue.

The molecular substrate underlying this IFN-α-associated change in MT is currently uncertain. In white matter, myelin dominates the MT exchange process (Kucharczyk et al., 1994, Odrobina et al., 2005), though in other neuronal structures processes such as inflammation (Stanisz et al., 2004), metabolic (Giulietti et al., 2012) and pH changes (Kucharczyk et al., 1994, Gillies et al., 2004, Louie et al., 2009) appear to play an important role. Previous data have linked regional changes in *k*_f_ to altered fluorodeoxyglucose (FDG) uptake (Giulietti et al., 2012, Harrison et al., 2015) suggesting a likely metabolic mediator. In brain, astrocytes account for approximately 50% of all glucose consumption (Bélanger et al., 2011, Chuquet et al., 2010). This increases further during glutaminergic activity (Porras et al., 2004) as is observed following IFN-α therapy (Haroon et al., 2014). Consequently, there is a sustained increase in lactate release from glia (Lin et al., 2010), a metabolite which contains both hydroxyl and carboxyl groups that are known to heavily influence MT exchange processes (Ceckler et al., 1992).

Changes in *k*_f_ are accompanied by co-localized changes in striatal T_2f_ (Harrison et al., 2015, Dowell et al., 2016). Although these MT metrics are sensitive markers for subtle microstructural change, they are not specific and are difficult to attribute to particular biological processes. Further characterization is possible with the aid of diffusion-weighted magnetic resonance imaging (MRI). In particular, we use neurite orientation dispersion and density imaging (NODDI) (Zhang et al., 2012) to model the diffusion-weighted signal within three distinct diffusion environments: unrestricted (isotropic diffusion) that broadly correspond to diffusion in the CSF, hindered (Gaussian displacement pattern) to extra-cellular, and restricted (non-Gaussian displacement pattern) to intracellular spaces. The NODDI model uses an orientation-dispersed cylindrical model where intracellular spaces are assumed to be cylinders of zero radius (restricted diffusion), while the extracellular spaces are characterized by hindered diffusion and modeled by a cylindrically symmetric diffusion tensor. The signal attributed to the intracellular spaces is assumed to be indicative of the neurite density. This neurite density index (NDI) ranges from 0 to 1 (NDI is typically lower in gray than white matter). A Watson distribution is then used to model the orientation distribution of the cylinders, which is quantified using the orientation dispersion index (ODI). Highly parallel white matter structures e.g. corpus callosum have ODI values close to 0 and areas like gray matter rich in multi-directional dendritic structure have values closer to 1.

We acquired multi-shell diffusion MRI in a group of 18 patients beginning IFN-α treatment for Hepatitis-C. Diffusion MRI was acquired at baseline (Mean 7 days prior to initiating therapy) and again 4 h after their first dose of IFN-α. Clinical assessments were completed at both scanning sessions and then again at 4, 8, and 12 weeks of treatment to quantify and characterize symptoms of fatigue and depression and relate these to acute changes in diffusion MRI. The striatum is known to be sensitive to chronic IFN-α (Harrison, 2017). Our primary aim was to determine whether striatal regions that demonstrate shifts in MT also show alterations in diffusion, specifically with NDI, which is sensitive to intracellular water diffusion and likely to be affected by changes in neuronal or possibly astrocytic processes and the ODI of these processes. We analyzed this in two ways: as a main effect of IFN-α and as a correlation with IFN-α-induced fatigue. We additionally investigated whether acute changes in NODDI metrics are also predictive about the later development of IFN-α-induced fatigue or mood change.

## Experimental procedures

### Participants

Eighteen individuals (mean age = 47 years, SD = 11 years, 13 male) with a diagnosis of hepatitis-C were recruited. All were fluent in English, aged between 18 and 64 years and fulfilled NICE guidelines for receiving pegylated IFN-α-based therapy. All participants had a baseline psychiatric evaluation of current mental state and previous psychiatric history, using the Mini Neuropsychiatric Inventory (MINI). Participants were excluded if they were receiving treatment for depression at study enrollment, had a history of psychotic illness, were not abstinent from substance misuse for at least 6 months, had HIV co-infection or any cause for liver disease other than hepatitis-C virus. The study was approved by the Cambridge Central National Research Ethics Committee. All subjects provided written informed consent. Seven participants recruited to this study also contributed to the qMT data reported in Dowell et al. (2016).

### Study design

The study utilized a prospective cohort design. Participants were evaluated at baseline (mean 7 days before treatment), 4 h after their first IFN-α injection and at weeks 4, 8, and 12 of IFN-α-based therapy. Fatigue and depressive symptoms were evaluated at each visit using a visual analog scale (fVAS) where participants marked their degree of fatigue on a 10-cm scale and the Hamilton Depression Rating Scale (HAMD) respectively. MRI followed by blood sampling, was repeated at baseline and 4 h after the first IFN-α injection.

### Behavioral analyses

Effects of IFN-α on fatigue and depression were analyzed in SPSS 23.0 (IBM Corp., Armonk, New York, USA) using repeated-measures analyses of variance and subsequent paired sample *t* tests or regression analyses, respectively. Mauchly’s sphericity test was performed, and results reported followed the Greenhouse–Geisser correction of degrees of freedom where appropriate.

### Cytokine analyses

Blood (20 mL) was drawn into Vacutainer tubes (Becton and Dickinson, Franklin Lakes, New Jersey, USA) containing ethylenediaminetetraacetic acid anticoagulant then centrifuged at 1250*g* for 10 min. Plasma was removed, aliquoted, and frozen at −80 °C before analysis. Plasma IFN-α was measured using high-sensitivity VeriKine ELISA (Human IFN Alpha Multi-Subtype ELISA Kit (TCM); PBL Assay Science, Piscataway, New Jersey). Interleukin-6 minimum detectable dose (MDD) = 0.039 pg/mL, tumor necrosis factor (TNF) MDD = 0.106 pg/mL, interleukin-1β MDD = 0.057 pg/mL, and interleukin-10 MDD = 0.09 pg/mL for the high-sensitivity Quantikine ELISAs (R&D Systems, Abingdon, United Kingdom) and interleukin-1 receptor antagonist MDD = 6.3 pg/mL for the Quantikine ELISA.

### MR imaging

MR imaging was performed on a Siemens Avanto (Siemens, Erlangen, Germany), equipped with a 1.5-T magnet and 32-channel phased-array receive-only head coil. Multi-shell diffusion-weighted data were acquired with single-shot, twice-refocused pulse gradient spin-echo echo planar imaging (voxel size 2.5 × 2.5 × 2.5 mm^3^, 60 axial slices, matrix size 96 × 96, field of view 240 × 240 mm^2^, repetition time = 8400 ms, echo time = 99 ms). Three *b*-value shells were acquired with *b* = 300, 800 and 2400 s/mm^2^ with 8, 30 and 60 non-colinear diffusion-weighted directions, respectively. Eleven images with no diffusion weighting (*b* ≈ 0) were acquired. Total acquisition time was 17 min.

### Image analysis

Diffusion data were first movement-corrected and eddy-current-corrected using the eddy_correct tool provided in FSL (FMRIB Software Library version 5.0.7, Oxford, UK). The MCFLIRT tool was used to quantify subject movement across the cohort during each imaging session. The NODDI model was fitted to the data using the NODDI toolbox (http://mig.cs.ucl.ac.uk/mig/mig/index.php/?n=Tutorial.NODDImatlab/) for Matlab (The MathWorks, Inc., Natick, MA, USA). Analysis took approximately 2 h per participant on a high-performance computing cluster with 128 compute cores (AMD ×86_64, 2.4 GHz) to yield whole-brain maps of NDI, isotropic water diffusion fraction (*V*_iso_) and ODI. Symmetric diffeomorphic image registration from Advanced Normalization Tools (ANTs, version 2.x; http://stnava.github.io/ANTs) was then used to deform non-diffusion-weighted (*b*_0_) images to a common MNI image space. These deformation matrices were then used to transform the whole-brain NODDI parameter maps to MNI space to permit group-level statistical comparison. Parameter maps were then smoothed with an 8-mm^3^ full width at half maximum Gaussian kernel.

Normalized parameter maps were then statistically analyzed using Statistical Parametric Mapping (SPM version 12, Wellcome Trust Centre for Neuroimaging, University College London, United Kingdom; http://www.fil.ion.ucl.ac.uk/spm/). Specifically, voxel-wise paired-sample *t*-tests were used to identify acute effects of IFN-α on regional NDI and ODI parameters and voxel-wide regression analyses to investigate the link between these MR-derived microstructural indices and fatigue.

### Regions of interest (ROI)

We defined four *a priori* ROIs for analyzing the effects of IFN-α: left and right striatum and left and right insula, matching those used in our previous studies (Harrison et al., 2015, Dowell et al., 2016). Masks were produced using the WFU Pickatlas (http://fmri.wfubmc.edu/software/pickatlas) then eroded where necessary, to avoid partial volume effects from cerebrospinal fluid (CSF). Mean NDI and ODI were calculated for each ROI then paired *t*-tests conducted using SPSS. Acute changes in fatigue and mood were correlated with mean NDI and ODI within each ROI using SPSS.

### Multiple comparisons

Whole-brain-corrected cluster significance was determined using Family-Wise Error (FWE) correction. Only clusters surviving a FWE correction *α* < 0.05 after thresholding at an uncorrected statistical threshold of *p* < 0.001 are reported for whole-brain analyses. Clusters surviving a FWE small-volume correction *α* < 0.05 after thresholding at an uncorrected statistical threshold of *p* < 0.001 are reported for each ROI.

## Results

### Inflammatory cytokine response to IFN-α

Initial IFN-α injection was associated with ∼31-fold increase in plasma IFN-α (from mean ± SE) (1.94 ± 1.31 pg/mL at baseline to 61.01 ± 13.73 pg/mL at 4 h, *t*_5_ = −4.24, *p* < 0.01). We also observed a fourfold increase in interleukin-6 (1.44 ± 0.30 pg/mL to 6.39 ± 1.25 pg/mL, *t*_12_ = −3.75, *p* = 0.003). Plasma TNF and interleukin-1β were not significantly altered at this time point (3.11 ± 0.68 pg/mL to 2.86 ± 0.58 pg/mL, *t*_12_ = 0.330, *p* = 0.747, and 0.0.335 ± 0.080 pg/mL to 0.333 ± 0.077 pg/mL, *t*_5_ = 0.021, *p* = 0.984), though there was a moderate increase in interleukin-1 receptor antagonist from 196.97 ± 40.13 pg/mL to 549.79 ± 172.45 pg/mL (*t*_12_ = −2.23, *p* = 0.045) and interleukin 10 from 0.782 ± 0.119 pg/mL to 1.76 ± 0.43 pg/mL (*t*_12_ = −2.52, *p* = 0.027) demonstrating a broader pro- and anti-inflammatory cytokine response to IFN-α.

### Psychological effects of IFN-α-based treatment

As we have previously reported, IFN-α treatment showed a strong effect on global fatigue (fVAS, *F*_1,17_ = 12.50, *p* = 0.003) increasing from 40.89 ± 6.36 to peak 64.28 ± 5.88 at 8 weeks. This increase in fatigue was rapid, with a moderate effect (*η*^2^ = 0.64) already observed at 4 h (*t*_17_ = 2.83, *p* = 0.015) demonstrating acute sensitivity to peripheral IFN-α. As previously reported, IFN-α-based therapy had a large effect on HAMD depression symptoms (*t*_17_ = 5.89, *p* < 0.001) with significant effects observed from 4 weeks until the end of treatment.

### MRI quality

No significant difference in subject movement was observed with imaging session (average root mean square displacement was 1.46 mm (at baseline) and 1.30 mm (at 4 h); *p* = 0.278) and no participants reported falling asleep during the scanning session. Signal-to-noise ratio (SNR) of the raw diffusion-weighted images was sufficient for the NODDI model (SNR >42 and >11 for *b* ≈ 0 and *b* = 2400 s/mm^2^ respectively).

### Acute effects of IFN-α on NODDI metrics

Unlike our previous finding using MT imaging, we identified no significant main effect of IFN-α within either our left or right striatal ROI or within our left or right insula ROI for either of the NODDI metrics (Table 1). Similarly, there was no significant effect of IFN-α at the whole-brain level.

**Table 1:** Paired *t*-test results for changes in NDI and ODI (from baseline to 4 h post first IFN-α injection) in the striatum and insula regions of interest

NODDI Metric	ROI	Baseline	4 h	Mean difference	*t*_(1,17)_	*p*
NDI	L striatum	0.4892	0.4834	−0.00583	−1.191	0.250
	R striatum	0.4908	0.4890	−0.00180	−3.99	0.695
	L insula	0.4538	0.4543	0.000572	0.143	0.888
	R insula	0.4620	0.4560	−0.00606	−1.697	0.108

ODI	L striatum	0.3902	0.3931	0.00290	1.721	0.105
	R striatum	0.3893	0.3867	−0.00259	−1.279	0.218
	L insula	0.4709	0.4654	−0.00551	−1.828	0.088
	R insula	0.4795	0.4741	−0.00533	−1.946	0.068

### Relationship with change in fatigue

**Fig. 1:**
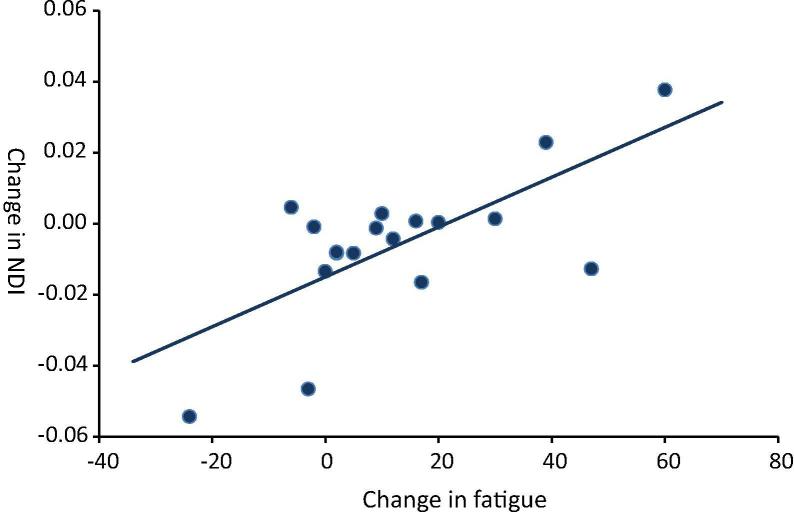
Plot of the correlation between acute change in NDI and acute change in fatigue in the left striatum (*R* = 0.69, *p* = 0.001).

To investigate this further, we next investigated whether this absence of an effect at the group level was driven by between-subject heterogeneity. Specifically, whether effects of IFN-α on striatal NODDI metrics emerged only in participants that developed significant IFN-α-induced fatigue. This analysis demonstrated a significant positive correlation between acute changes in NDI and fatigue in our (left) striatal and, to a lesser extent our (left) insula ROI (Fig. 1; Table 2). Furthermore, the effect size of this association was modestly large (*R*^2^ = 0.52 and *R*^2^ = 0.46 respectively). When accounting for the change in mood by regressing out change in HAMD score the relationship between NDI and fatigue was preserved (*R* = 0.682; *p* = 0.002 (acute), *R* = 0.607; *p* = *p* = 0.008 (at 4 weeks) and *R* = 0.673; *p* = 0.002 (at 8 weeks)). This indicates that changes in fatigue and HAMD score were largely independent. Similar to our MT findings, acute changes in NDI within the left striatum but not the insula additionally predicted the magnitude of fatigue experienced 4 and 8 weeks later. There was no significant correlation between acute fatigue and fatigue at 4 weeks (*p* = 0.055) and 8 weeks (*p* = 0.111).

**Table 2:** Pearson’s correlation coefficient (*R*) and statistical significance (*p*) of correlations between acute change in NODDI metrics (baseline to 4 h post first IFN-α injection) with change in fatigue at 5 h (acute), 4 weeks, and 8 weeks post-onset of IFN-α treatment (p < 0.05 shown in bold)

Correlation with Δ fVAS	Statistic	L striatum	R striatum	L insula	R insula
Δ NDI (4 h)	*R*	0.692	0.419	0.529	0.348
	*p*	**0.001**	0.083	**0.024**	0.157
Δ NDI (4 weeks)	*R*	0.664	0.398	0.128	0.297
	*p*	**0.003**	0.102	0.612	0.231
Δ NDI (8 weeks)	*R*	0.651	0.367	0.163	0.339
	*p*	**0.003**	0.134	0.517	0.169

Δ ODI (4 h)	*R*	0.068	0.180	0.100	0.196
	*p*	0.789	0.474	0.694	0.437
Δ ODI (4 weeks)	*R*	−0.201	0.121	0.268	0.498
	*p*	0.425	0.671	0.283	0.035
Δ ODI (8 weeks)	*R*	−0.248	−0.019	0.269	0.467
	*p*	0.320	0.941	0.281	0.051

**Fig. 2:**
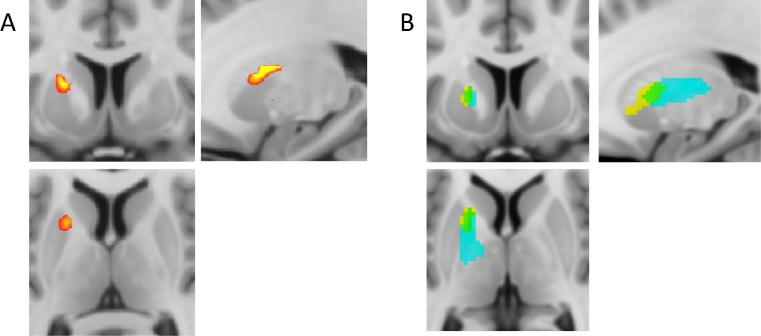
Voxel-wise correlation analysis of acute change in NDI (baseline to 4 h) with acute change in fatigue (baseline to 4 h). (A) 80-mm field of view focused on the striatal regions of interest. (B) Location of changes in qMT previously reported in Dowell et al., 2016. Data illustrated at an uncorrected threshold of *p* < 0.001.

In contrast, ODI did not significantly correlate with fatigue at any time-point. Similar to our prior MT analysis, there was no significant association between changes in striatal (or insular) NDI or ODI and change in mood score measured with HAMD. Whole-brain analysis confirmed the significant positive correlation between left striatum increases in NDI and development of fatigue at 4 h post injection (Fig. 2). It also identified a number of additional clusters elsewhere showing a similar positive correlation with fatigue (Table 3). Of note, no region showed a negative correlation between changes in NDI and fatigue and no significant correlation was observed between change in ODI and fatigue.

**Table 3:** Brain areas with positive correlation of acute change in NDI with acute fatigue. ^*^Indicates *a priori* regions of interest. FWE-corr = cluster wise family-wise error-corrected

Cluster location	Cluster volume (mL)	*Z*-score	MNI co-ordinates (mm)	*p*(FWE-corr)
Left striatum^*^	1.4	4.33	−22,0,16	0.084
Right insula^*^	1.8	4.19	36,12,8	0.037
Precentral gyrus	2.0	4.48	−14,−30,50	0.023
Occipital cortex	19.4	4.54	48,−68,24	<0.001

## Discussion

IFN-α treatment of hepatitis C is characterized by a rapid induction of fatigue that starts within hours of the first injection then typically increases throughout treatment (Dowell et al., 2016). This motivationally impairing effect of IFN-α has been localized to changes in striatal function across a broad range of imaging modalities. For example, chronic IFN-α administration is associated with increases in striatal ^18^FDG uptake (Capuron et al., 2007), an increase in glutamate/creatine ratio (Haroon et al., 2014); and increased ^18^fluorodopa uptake (Capuron et al., 2012). Recently, we have also demonstrated that IFN-α acutely impairs striatal MT, specifically a significant increase in forward magnetization transfer rate (*k*_f_) and a simultaneous decrease in the transverse relaxation time of the free water component (T_2f_). In this study these MT metrics were also predictive of the development of fatigue 4 weeks later into treatment. More constrained changes were also detected in the insula, though these did not correlate with fatigue.

Here we used NODDI in an overlapping cohort of patients to provide a more complete and deeper understanding of the subtle changes in brain microstructure induced by IFN-α. Of particular interest was the ability of NODDI to identify changes to the intracellular water spaces within tissue. While NDI does not provide a one-to-one mapping of neurite volume fraction, it is sensitive to changes within the intracellular component (Grussu et al., 2017). Therefore, it is plausible that NDI provides an index for inflammatory processes such as cellular swelling. In rodent studies peripheral inflammation has been associated with both an increase in astrocytic (specifically microglial) cell body size and a reduction in microglial processes (Rivest, 2009). NODDI is potentially sensitive to both of these processes. However, it is important to note that a number of complex factors influence the signal intensity in diffusion-weighted imaging. Not least, subtle changes in the relaxation properties of discrete microstructural compartments will also manifest as changes in NDI. Indeed, our prior qMT study on a partially overlapping cohort revealed a subtle decrease in T_2f_ in the left striatum (Dowell et al., 2016). However, we observed no significant correlation between changes in T_2f_ and the changes to NDI (data not shown). This suggests that the T_2_ changes in the intracellular component are absent or too small to effect a measurable change in NDI.

Interestingly, our current study revealed that, although the striatum and insula showed no significant differences in either NDI or ODI pre- and post-injection, there was a strong positive correlation between increases in striatal NDI and the experience of fatigue both acutely and at 4 and 8 weeks later into treatment. These data suggest that changes in intracellular water (possibly within microglia) play a role in the emergence of IFN-α-induced fatigue. They also reinforce the observation that the striatum is a key brain area targeted by IFN with implications for impaired motivation. In keeping with our previous qMT findings, there was no correlation between changes in any of the NODDI metrics and acute or later change in mood measured using the HAMD questionnaire. This further strengthens the notion that distinct mechanisms underpin the action of IFN-α on fatigue and depressive symptoms.

Our previous qMT findings were striking in that striatal changes were predominantly lateralized to the left hemisphere. It is therefore compelling that the same lateralization pattern was also observed in the present study, with changes in NDI again correlating with fatigue most strongly in the left striatum (with only trend-level significance on the right) (Fig. 2). This left-sided laterality appears to be a recurring theme in studies of inflammatory challenge across a broad range of imaging modalities. For example, it has been observed in an FDG PET study linking changes in glucose uptake in the left ventral striatum to fatigue (Capuron et al., 2007) and a MR spectroscopy study that revealed that left but not right IFN-α-induced changes in striatal glutamate/creatine ratio correlated with associated changes in motivation.

The modest sample size (18 participants, each scanned twice) could be considered a potential limitation of this study. However, our use of a within-subject study design serves to partially mitigate this by affording improved power and sensitivity over cross-sectional or between-group studies. Recently, the NODDI model has also attracted some criticism recently regarding the use of a number of assumptions that, if violated, can result in either a positive or negative bias in the NODDI parameters (Jelescu et al., 2016, Lampinen et al., 2017). While the model has some limitations, it is important to acknowledge that the same is true for all models that attempt to use the microstructure from diffusion-weighted imaging data. Furthermore, NODDI provides improved characterization of tissue microstructure compared to the more simplistic models such as diffusion tensor imaging.

To conclude, we used NODDI to probe subtle changes to tissue microstructure associated with IFN-α. This revealed a strong correlation between changes in NDI and the level of fatigue experienced both acutely and longer into the treatment regimen. While this represents a complementary quantitative MR imaging approach to qMT, it is important to note that these techniques are completely independent and use different imaging data and different analysis pipelines. As a consequence, it is compelling that both qMT and NODDI implicate the striatum so strongly in the development of fatigue in patients treated with IFN-α.
